# Zwitter-Ionic Polymer Applied as Electron Transportation Layer for Improving the Performance of Polymer Solar Cells

**DOI:** 10.3390/polym9110566

**Published:** 2017-11-01

**Authors:** Qiaoyun Chen, Zhendong Li, Bin Dong, Yi Zhou, Bo Song

**Affiliations:** College of Chemistry, Chemical Engineering and Materials Science, Soochow University, Suzhou 215123, China; chenqiaoyun1995@163.com (Q.C.); lizhendong@zoho.com (Z.L.); bdong@suda.edu.cn (B.D.)

**Keywords:** PSBMA, betaine, zwitter-ionic polymer, ETL

## Abstract

A zwitter-ionic polymer poly (sulfobetaine methacrylate) (denoted by PSBMA) was employed as an electron transportation layer (ETL) in polymer solar cells (PSCs) based on poly(3-hexylthiophene) (P3HT):[6,6]-phenyl-C_61_-butyric acid methyl ester (PC_61_BM). PSBMA is highly soluble in trifluoroethanol, showing an orthogonal solubility to the solvent of the active layer in the preparation of multilayered PSCs. Upon introduction of PSBMA, the short circuit current and as a consequence the power conversion efficiency of the corresponding PSCs are dramatically improved, which can be because of the relatively high polarity of PSBMA compared with the other ETLs. This study demonstrated that zwitter-ionic polymer should be a competitive potential candidate of ETLs in PSCs.

## 1. Introduction

Polymer solar cells (PSCs) with a bulk heterojunction (BHJ) structure have attracted extensive research attention owing to advantages, such as light-weight, low cost, flexibility, and large-area fabrication [1,2]. Recently, the power conversion efficiencies (PCEs) of single-junction PSCs have reached 13% [3,4,5,6], owing to the molecular design of photoactive materials [3,7,8,9,10,11,12], interface engineering [13,14,15,16], and improvement of device configurations. Although the design of new photoactive materials plays a key role in this concern, the steps for exploration of high efficient interfacial materials that can facilitate the charge collection and transportation have never ceased. For PSCs, the interfacial materials are demanded to have good wetting processing ability and solvent orthogonal property with the solvent used to dissolve the photoactive materials. In this regard, compared with conventional inorganic electron transportation layers (ETLs), such as Ca and LiF etc. [17], the water-/alcohol-soluble polymers (polyfluorene derivatives [18,19], polyethyleneimine [20,21,22,23], ethoxylated polyethyleneimine [24,25] etc.), and small organic molecules [3,26,27,28] show their priority in fabrication of low-cost and large-area PSCs [29]. The inorganic metal oxides (e.g., ZnO [30,31,32,33], TiO_x_ [34,35,36,37], etc.), metal salts (CsF [38], Cs_2_CO_3_ [39], etc.), and self-assembled monolayers are successfully applied to improve the performance of PSCs. However, most of these inorganic ETLs showed poor interfacial contact with organic photoactive layer [40], which may hinder the effective charge extraction. In addition to the above-mentioned aspects, for organic interfacial materials, the suitable dipole moment is also an important factor that may affect the work function of the electrodes, and thus influence the resulting performance of the solar cell devices.

To meet all the aforementioned properties, organic interfacial materials with functionalities, such as phosphonate [41], ethylene oxide [42,43], amino or ammonium [44,45], etc. have been widely studied as ETLs. It has been proven that the introduction of these materials can indeed lower the work function of the adjacent electrode, and thus lead to the improvement of the corresponding solar cell devices. For this purpose, zwitter-ionic polymers that contain approximately twice as many polar groups compared with the typical ionic molecules, can possibly give higher surface dipoles, and thus be favorable for the electron transportation [26,46,47,48].

Herein, a solution-processable, electronically neutral zwitter-ionic polymer poly (sulfobetaine methacrylate) (denoted by PSBMA) was employed as the ETL in PSCs. Since P3HT was widely studied as a model material in PSCs, herein we also employed P3HT:PC_61_BM as the active layer. It was anticipated that the sulfobetaine moiety on the side chain should provide a strong permanent moment, which can reduce the work function (WF) of cathode electrode and then alleviate the interfacial energy barriers. The results indicate that PSBMA provides orthogonal solubility in the fabrication of multi-layered solar cells. Under optimized conditions, a PCE of 3.67% was achieved when using PSBMA as ETL, presenting a notable improvement compared with that (3.49%) of the devices without PSBMA.

## 2. Materials and Methods

### 2.1. Materials

[2-(methacryloyloxy)ethyl]dimethyl-(3-sulfopropyl)ammonium hydroxide, 4,4-azobis(4-cyanovaleric acid) (ACVA), and 4-cyano-4-(thiobenzoylthio) pentanoic acid were purchased from Alfa Aesar (China) Chemicals Co., Ltd. (Shanghai, China). 2,2,2-trifluoroethanol (TFE) was purchased from J&K Technology Co., Ltd. (Beijing, China). The PSBMA was synthesized according to the reported literature [49,50,51]. The PEDOT:PSS solution was purchased from Heraeus Precious Metals GMBH & Co. KG (Leverkusen, Germany). P3HT and PC_61_BM were purchased from 1-Material Co., Ltd. (Dorval, QC, Canada) and Solarmer Materials Inc. (Beijing, China), respectively. Al was acquired from Zhong Nuo Advanced Material Technology Co., Ltd. (Beijing, China).

### 2.2. Fabrication of Devices

The PSCs were fabricated with a configuration of ITO/PEDOT:PSS/P3HT:PC_61_BM/ETL/Al. The ITO-coated glass (10 Ω per square) was cleaned by sequential ultrasonification in water containing the dish washing liquid, deionized water, acetone, ethanol and isopropanol twice each solvent and 15 min each time, and then treated with ultraviolet-ozone by a UVO cleaner (Jelight Company, Inc., 2 Mason, Irvine, CA, USA) for 20 min. A PEDOT:PSS layer (~40 nm) was spin-coated onto the cleaned ITO substrates at 5000 rpm for 40 s, and the substrates were annealed at 150 °C for 15 min in air. Then, an *o*-chlorobenzene solution of P3HT:PC_61_BM blend (1:1 *w*/*w*, total concentration of 40 mg/mL) was spin-coated onto the PEDOT:PSS at 900 rpm for 30 s. Under the above conditions, the thickness of the P3HT:PC_61_BM blend were controlled to ~230 nm. The TFE solution of PSBMA with a concentration of 0.25, 0.5, and 0.75 mg/mL was spin-coated onto the active layer at a speed of 4500 rpm for 45 s, respectively. The Ca layer (20 nm) and the Al electrode (80 nm) was thermally evaporated atop of PSBMA with a shadow mask to define the effective area of 0.04 cm^2^ under a pressure of 2 × 10^−4^ Pa.

### 2.3. Measurement and Characterization

Nuclear Magnetic Resonance (NMR) spectrum was measured on Bruker AV-500 MHz spectrometer (Bruker, Santa Barbara, CA, USA). Gel permeation chromatography measurement (GPC) was carried out in TFE with 0.02 mol/L sodium trifluoroacetate at 40 °C using an Agilent 1200 system equipped with an isocratic pump operated at 1 mL/min, a degasser, an autosampler, one 50 mm × 8 mm PSS PFG guard column (Polymer Standards Service), three 300 mm × 7.5 mm PSS PFG analytical linear M columns with particle size of 7 μm (Polymer Standards Service) calibrated against poly(methyl methacrylate) (PMMA) standards, and an Agilent 1200 refractive index detector (Agilent, Anaheim, CA, USA). Electrochemical cyclic voltammetry (CV) was performed on a Zahner Ennium IM6 Electrochemical Workstation with a glassy carbon disk, Pt wire, and Ag/Ag^+^ electrode as the working electrode, counter electrode, and reference electrode, respectively. The tetra-*n*-butylammoniumhexafluoro-phosphate (*n*-Bu_4_NPF_6_, 0.1 mol/L in acetonitrile) as the supporting electrolyte. The ferrocene/ferrocenium (Fc/Fc^+^) was used as an internal standard, which was assigned an absolute energy of −4.8 eV vs. vacuum level. The morphologies of active layer and ETL surfaces were characterized by Atomic Force Microscope (AFM) on a Multimode 8 microscope (Bruker, Santa Barbara, CA, USA) in air using ScanAsyst-Air probes. The force constant was 0.4 N/m. The set point was 0.08 V. The scan rate was 0.977 Hz. The thicknesses of the films were recorded with a spectroscopic ellipsometer (M-2000 V, J.A. Woollam Co., Lincoln, NE, USA). The *J*-*V* curves were measured in a glovebox with an SS-F5-3A solar simulator and a Keithley 2400 source meter unit under standard Air Mass 1.5 Global (AM 1.5 G) (100 mW cm^−2^) illumination calibrated by a standard Si solar cell (SRC-2020, Enli Technology Co., Ltd., Taiwan) and when testing there was no mask. The external quantum efficiency (EQE) data were recorded on a QE-R3011 (Enli Technology Co., Ltd., Taiwan), where the light intensity was calibrated by a standard Si solar cell (RC-S10-A, Enli Technology Co., Ltd., Taiwan) certified by Taiwan Accreditation Foundation (TAF).

## 3. Results and Discussion

### 3.1. Synthsis of the PSBMA

**Scheme 1:**
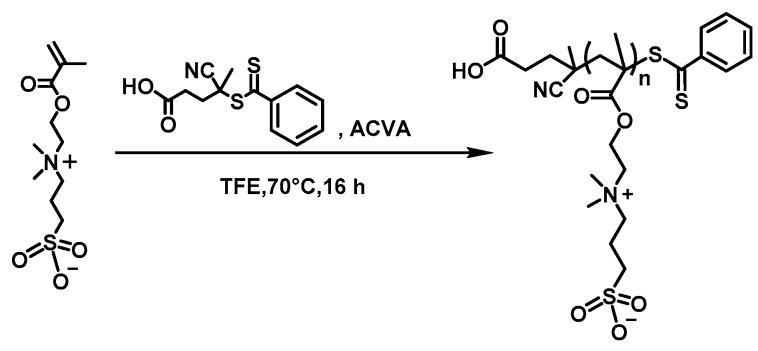
Synthetic route of poly (sulfobetaine methacrylate) (PSBMA).

**Figure 1:**
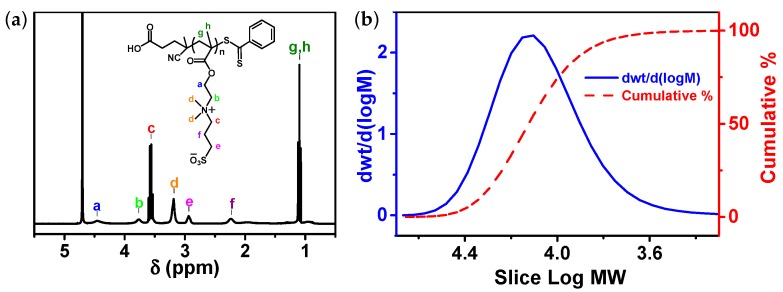
(**a**) ^1^H-NMR spectrum and (**b**) Gel permeation chromatography measurement (GPC) curves of PSBMA, where *wt* is weight, *M*_w_ is molecular weight.

The synthetic route of the target compound PSBMA is shown in Scheme 1, which mainly referred to the work of Zachariah et al. [49,50,51], using the reversible addition-fragmentation chain transfer (RAFT) polymerization. Herein, methacryloxyethyl sulfobetaine, 4-cyano-4-(thiobenzoylthio) pentanoic acid, ACVA and TFE were used as the monomer, chain transfer agent, initiator, and solvent, respectively. The product is a fine pink powder, showing a good solubility in TFE and water but a poor solubility in methanol and ethanol. Because of its unique solubility, TFE can be used as a solvent to avoid damaging the active layer during the spin-coating process. As shown in Figure 1, the resulting product was confirmed by ^1^H-NMR spectra and GPC. The ^1^H-NMR spectra shows the resonances for the –COOCH_2_^−^ group at δ 4.56 ppm (labeled as protons a), for the –CH_2_N^+^(CH_3_)_2_CH_2_^−^ group at δ 3.86, 3.28, 3.66 ppm (labeled as protons b, d and c, respectively), for –CH_2_^−^ group at δ 2.33 ppm (labeled as protons f), for –CH_2_SO_3_^−^ group at δ 3.02 ppm (labeled as protons e) and for the –CH_2_CCH_3_^−^ group at δ 1.04~1.21 ppm (labeled as protons g and h, respectively). As listed above, the chemical shifts correspond to the protons in different chemical environment of the target molecule, and the most of the peak became broad and the coupling information were not differentiable. These results indicate the compound we have synthesized should be the polymer we designed. The ^1^H-NMR data are also in accordance with the literature [52], further confirming the chemical structure of the target polymer. The result of GPC showed that the number average molecular weight (*M*_n_) is 11.4 kDa, and weight average molecular weight (*M*_w_) is 13.8 kDa. The polydispersity index (PDI) is 1.2, implying that the product has a relatively narrow molecular weight distribution.

### 3.2. The Thickness Control of the PSBMA Films

**Figure 2:**
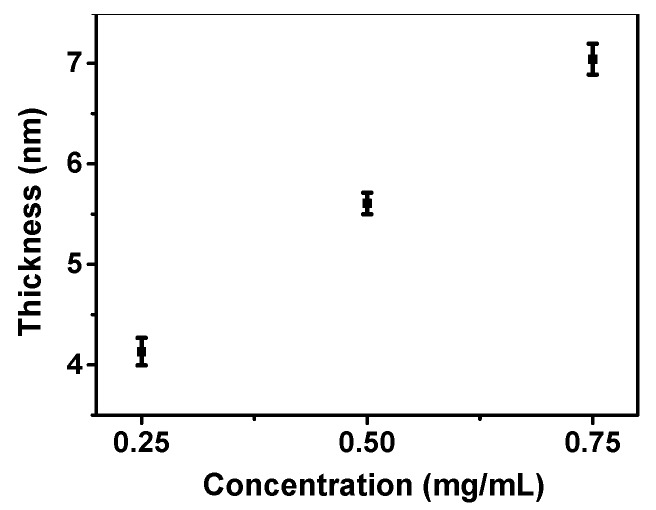
The thickness versus the corresponding concentration of PSBMA. The films were prepared by spin-coating at 4500 rpm for 45 s.

The thicknesses of spin-coated films mainly depend on the solution concentration, spin speed, and time. In order to obtain a parallel comparison, herein the spin speed and time were fixed to 4500 rpm and 45 s, respectively. The concentration of the PSBMA solution was taken as variables to investigate the thickness change, and the silicon wafers were employed as substrates. After being prepared on silicon wafers, the PSBMA films were annealed at 80 °C for 15 min, and then the thicknesses of them was determined on an ellipsometer. As shown in Figure 2, the thicknesses of the films increased with the concentration of the corresponding solutions. Through varying the concentrations from 0.25, 0.5, to 0.75 mg/mL, the film thicknesses of approximately 4.1, 5.6, and 7.0 nm were obtained, respectively.

### 3.3. Morphologies of P3HT:PC_61_BM and PSBMA@P3HT:PC_61_BM

**Figure 3:**
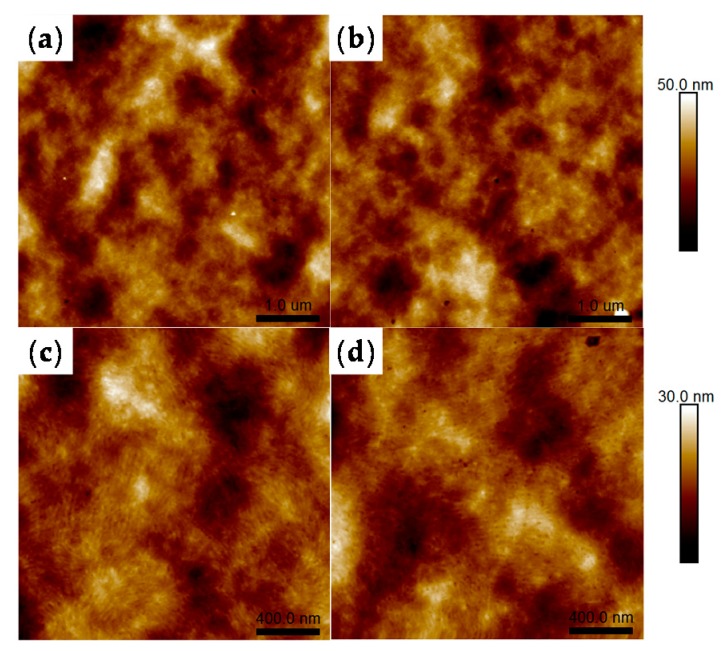
Atomic Force Microscope (AFM) height images of (**a**,**c**) P3HT:PC_61_BM and (**b**,**d**) PSBMA@P3HT:PC_61_BM. Here the thickness of PSBMA was 5.6 nm. The scanning areas of (**a**,**b**) and (**c**,**d**) are 5 × 5 µm^2^ and 2 × 2 µm^2^, respectively.

The surface morphology and aggregation state of the interlayer have great effect on the device performance and an active layer with an inner film morphology fulfills the requirements of high-performance solar cells [53,54]. In addition, two more specific variables drive us to investigate the morphology of the PSBMA. (1) TFE is not an often-used solvent for preparation of thin films on P3HT:PC_61_BM active layer; (2) the highly polar PSBMA due to the ionic feature might be a problem on spreading at the apolar surface of P3HT:PC_61_BM. Figure 3 shows the AFM height images of the P3HT:PC_61_BM and PSBMA@P3HT:PC_61_BM. In the previous publications [55,56], the effect of solvent on the morphology of P3HT:PCBM blend films was investigated by different methods, such as AFM and grazing-incidence wide-angle X-ray scattering. The surface morphology measured by AFM can also reflect the inner structure of the films. Herein, the images clearly show the phase separation of the active layer. After being covered by 5.6 nm thick PSBMA film, the surface morphology did not change too much, and the phase separation of the active layer can still be observable. Through these images, we can conclude that PSBMA can spread uniformly on the P3HT:PC_61_BM blend film, and the TFE solvent has very little influence on the morphology of the active layer.

### 3.4. Electrochemical Properties 

**Figure 4:**
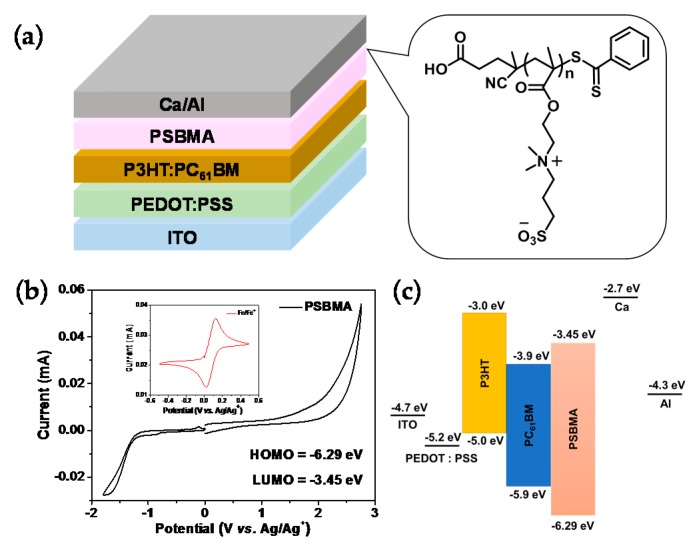
(**a**) Schematic illustration of the device structure of PSCs adopted in this study. (**b**) The cyclic voltammetry (CV) curve of PSBMA. (**c**) Energy level diagram of each component used in the PSCs.

In this study, we adopted the conventional solar cell structure (i.e., using ITO as anode (as shown in Figure 4a), and PSBMA was inserted between the active layer and the cathode) to investigate the charge transportation ability of PSBMA. The devices without PSBMA layer were also fabricated in parallel conditions and adopted as control. One of the key issues of interfacial layers is the energy level alignment. Herein, the energy levels of PSBMA were determined by cyclic voltammetry (CV) as shown in Figure 4b, in which the small image showed the CV curve of Fc/Fc^+^ under the same experimental conditions. From the CV curve, the highest occupied molecular orbital (HOMO) level (*E*_HOMO_) and the lowest unoccupied molecular orbital (LUMO) level (*E*_LUMO_) of PSBMA were estimated to be −6.29 and −3.45 eV, respectively. The energy levels of each component in the PSCs are illustrated in Figure 4c. It is clearly shown that PSBMA should be suitable to be an ETL between the active layer and cathode. In addition to the energy level alignment, the high polarity of PSBMA was also expected to improve the transportation of electron and reduce the interfacial charge recombination. This assertion is in accordance with the previous publications [26,57,58]. For example, the highly polar –SO_3_^−^ and amine are both responsible to the transportation of charge carriers around the electrodes [26].

### 3.5. Photovoltaic Properties 

**Figure 5:**
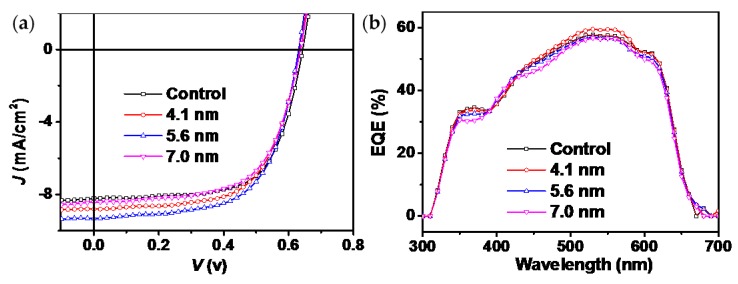
(**a**) Density-voltage (*J*-*V*) and (**b**) external quantum efficiency (EQE) curves of PSCs without and with PSBMA ETLs. The thickness of PSBMA varied as 4.1, 5.6, and 7.0 nm.

To investigate the effect of the PSBMA interlayer on the performance of the devices, the photovoltaic properties of the corresponding PSCs was investigated under simulated AM 1.5 G illumination with an intensity of 100 mW/cm^2^. The current density-voltage (*J*-*V*) curve and the corresponding external quantum efficiency (EQE) curve of the PSC devices are shown in Figure 5. The detailed performance parameters of the corresponding devices are summarized in Table 1. The control device reached a PCE of 3.49%, with the short-circuit current density (*J*_sc_) of 8.21 mA/cm^2^, an open-circuit voltage (*V*_oc_) of 0.64 V and a fill factor (FF) of 65.9%. Upon insertion of PSBMA interlayer, *J*_sc_ of the device was clearly improved. When the thickness of PSBMA layer was 5.6 nm, a highest PCE was achieved up to 3.67% with a *J*_sc_ of 9.32 mA/cm^2^, a *V*_oc_ of 0.63 V, and a FF of 62.5%. The increase of *J*_sc_ can also be confirmed by the integrated current density (*J*_int_) obtained from the corresponding EQE curves. These two groups of values are also comparable, indicating that the *J*_sc_s from *J*-*V* curves should be reliable.

## 4. Conclusions

In this study, the classical P3HT:PCBM-based PSCs was employed as a model system to demonstrate the possibility of using zwitter-ionic polymer as ETLs in PSCs. Herein, a betaine-based polymer (i.e., PSBMA) was synthesized and used as a zwitter-ion material. Owning to the high polarity, PSBMA showed a clear improvement effect on the current density of the resulting PSC devices, and hence the total PCE was also enhanced. Although PSBMA has high polarity nature, the film can spread well on the apolar P3HT:PC_61_BM surface, and the rarely used TFE solvent have little damage on the surface morphology of P3HT: PC_61_BM. This work may offer a new strategy to design ETL materials for highly efficient PSCs.

## Figures and Tables

**Table 1:** Detailed parameters of PSC devices with/without PSBMA ETLs.

ETL	V*oc* (V)	J*sc* (mA/cm^2^)	FF (%)	PCE (%)
Ca	0.64 ^a^ (0.63 ± 0.01) ^b^	8.21 (8.04 ± 0.17)	65.9 (64.8 ± 0.7)	3.49 (3.31 ± 0.12)
4.1 nm	0.64 (0.63 ± 0.01)	8.81 (8.52 ± 0.20)	64.7 (63.4 ± 1.0)	3.51 (3.43 ± 0.05)
5.6 nm	0.63 (0.63 ± 0.00)	9.32 (8.99 ± 0.19)	62.5 (60.7 ± 1.5)	3.67 (3.44 ± 0.15)
7.0 nm	0.64 (0.63 ± 0.01)	8.43 (8.29 ± 0.01)	64.1 (62.9 ± 0.7)	3.37 (3.29 ± 0.05)

^a^ Optimal device; ^b^ Average value obtained from six devices.
